# A relapsed/refractory acute promyelocytic leukemia achieving complete response after chemotherapy with venetoclax

**DOI:** 10.1097/MD.0000000000046365

**Published:** 2025-12-12

**Authors:** Xiaolin Zhang, Shuhong Hao, Chunmei Hu, Rongfeng Qu

**Affiliations:** a Department of Oncology and Hematology, The Second Hospital of Jilin University, Changchun, Jilin, China.

**Keywords:** acute promyelocytic leukemia, case report, complete response, Relapsed and refractory, venetoclax

## Abstract

**Rationale:**

The vast majority of patients newly diagnosed with acute promyelocytic leukemia (APL) can achieve complete response (CR) through induction therapy that includes all-trans retinoic acid (ATRA), and subsequently achieve molecular response after consolidation therapy. Relapses are quite rare after achieving molecular response. Nonetheless, 5% to 10% of APL patients who receive ATRA plus anthracycline chemotherapy may experience relapse. Compared to newly diagnosed APL patients, the treatment of relapsed/refractory APL remains a challenge due to its rarity. This article reports a case of an APL patient with multiple relapses who achieved complete response following chemotherapy with venetoclax (VEN) in combination with idarubicin and cytarabine.

**Patient concerns:**

The patient is a 58-year-old male who was definitively diagnosed with acute promyelocytic leukemia 20 years ago. One month ago, he presented with gum bleeding. A bone marrow examination revealed that abnormal promyelocytes accounted for 58.0%, and a complete blood count indicated pancytopenia.

**Diagnoses:**

The patient has a definitive diagnosis of relapsed/refractory APL, complicated by a DNMT3A mutation and complex karyotype, indicating a poor prognosis.

**Interventions:**

Complete response was achieved after the salvage chemotherapy, specifically: VEN 100 mg day 4, 200 mg day 5, 400 mg days 6–11; Idarubicin 20 mg days 1–2; Cytarabine 100 mg bid days 1–5.

**Outcomes:**

After VEN-based therapy, the patient exhibited no symptoms of leukemia and had achieved complete response.

**Lessons:**

For relapsed and refractory APL, relevant drug resistance gene monitoring should be carried out. Some relapsed and refractory APL patients who do not respond to conventional treatment are at risk of death. We report a successful case, the regimen of VEN targeted therapy combined with chemotherapy still holds promise for the treatment of future relapsed/refractory APL.

## 1. Introduction

Acute promyelocytic leukemia, a distinct type of acute myeloid leukemia (AML) characterized by hemorrhagic phenomena and decreased blood cell counts, accounts for approximately 10% to 15% of diagnosed AML cases.^[1]^ According to the latest research, the combination of ATRA and ATO has become the standard treatment protocol for APL and has achieved excellent therapeutic outcomes. Notably, although the cure rate of typical APL is close to 100%, some patients may still experience recurrence, and treatment remains a significant challenge, particularly for patients with atypical APL, refractory or relapsed APL. Relevant literature suggests that VEN-based therapy is a viable salvage therapy for relapsed,^[2–4]^ refractory nonpremature granulocytic, but its application in APL has not been reported. In this paper, we present a case of relapsed/refractory acute promyelocytic leukemia in a patient who had multiple relapses after the first response with induction therapy with ATO, ATRA, epirubicin, and cytarabine, during which several induction therapies failed, and then achieved complete response after salvage chemotherapy with combining VEN, idarubicin, and cytarabine.

## 2. Case presentation

**Figure 1.**
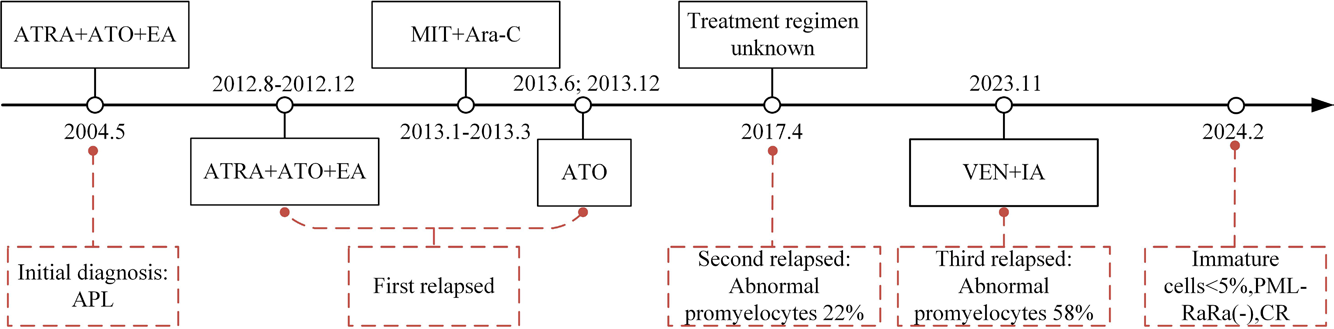
Patient’s treatment course. EA = epirubicin and cytarabine, MIT = mitoxantrone, Ara-C = cytarabine, IA = idarubicin and cytarabine.

A 58-years-old man patient was admitted to local hospital in MAY 2004. He was diagnosed with PML-RARA-positive acute promyelocytic leukemia, based on bone marrow cytology, immunophenotypic analysis by flow cytometry, molecular analysis and karyotype analysis. He received one courses of induction therapy with ATRA and ATO combined with chemotherapy (epirubicin and cytarabine) attaining complete response. In August 2012, the patient returned to local hospital for feeling feverish. According to results of Bone-marrow smear, which showed increased blasts, he experienced a relapse, achieving CR after treated induction therapy with the original induction regimen, followed by 3 cycles of therapy (mitoxantrone and cytarabine), and Maintenance therapy with ATO. Patient’s treatment course is shown in Figure 1.

**Figure 2.**
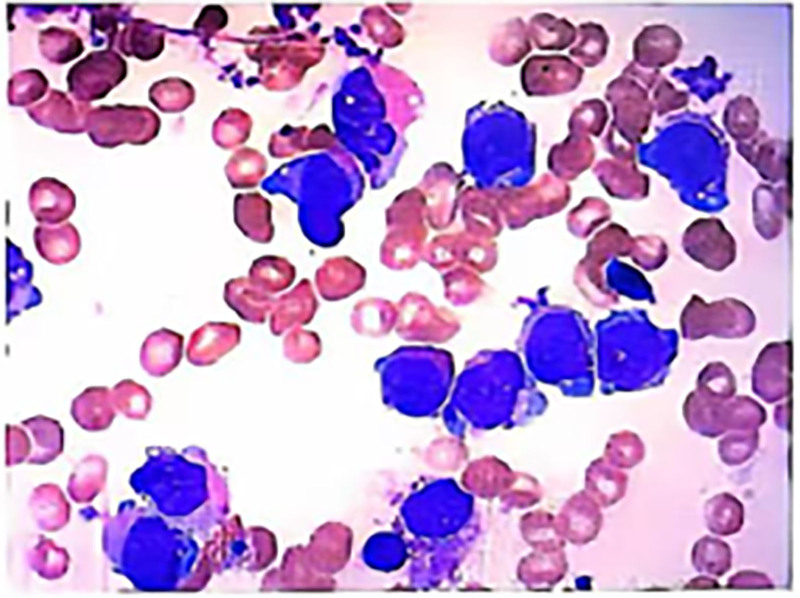
Bone marrow findings before VEN. VEN = venetoclax.

In April 2017, the patient relapsed again, and a bone marrow examination revealed “22% blasts and promyelocytes,” after which he was given systemic treatment. In November 2023, the patient experienced gum bleeding without any apparent cause. A bone marrow examination revealed: “Abnormal promyelocytes accounted for 58.0%.” A complete blood count showed pancytopenia, and the patient also had a fever with a maximum body temperature of 39.3°C. A follow-up bone marrow examination indicated: abnormal promyelocytes accounted for 21% (Fig. 2). Concurrently, relevant genetic testing was performed: PML/RARA (L) positive. Myeloid tumor-related gene mutation testing: WT1 11p13 NM-024426 exon8 p.F437* 3.8%. WT1 11p13 NM-024426 exon7 p.S386Cfs*74 0.8%. DNMT3A 2p23.3 NM-022552 exon23 p.R882H 2.2%. Flow cytometry results indicated that abnormal cells accounted for 22.95% of the total nucleated cells, and this cell population expressed: CD13, CD33; partially expressed CD117; did not express CD11b, CD15, CD16, CD19, CD34, CD9, CD123, HLA-DR. PML/RARA quantification: bcr-1 (L type) copy number 99,182, ABL1 gene copy number 255,163, bcr-1/ABL1 38.87%. Karyotype: 46, XY; i(7) (p10); t(8;14)(p21;q13); t(15;17)(q24;q21).

**Figure 3.**
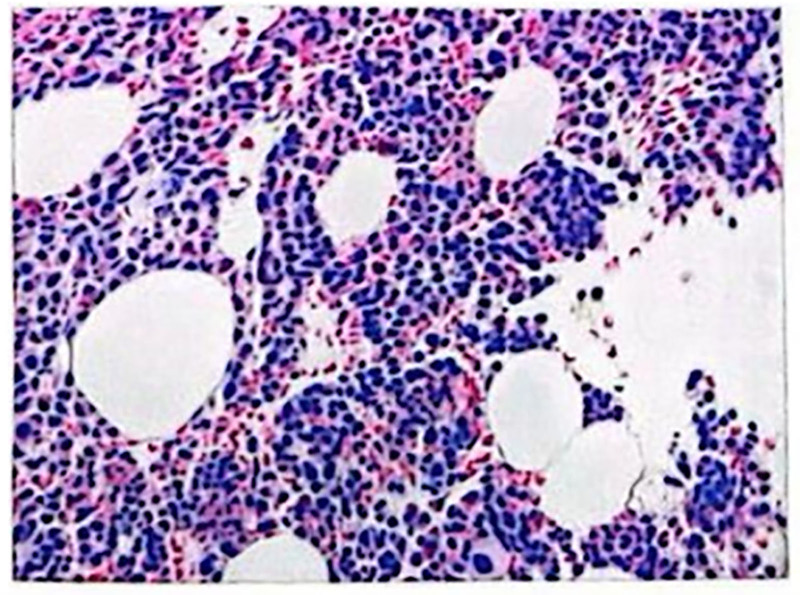
The laboratory indicators change trend of the patient. During chemotherapy, the patient experienced grade 4 myelosuppression and developed an infection. we provided active transfusion support. On day 20 the white-cell count increased to 4.8*10^9^/L and transfusions were discontinued. On the 21st day, a peripheral blood smear showed no evidence of immature cells.

The patient has a definitive diagnosis of relapsed/refractory APL, complicated by a DNMT3A mutation and complex karyotype, indicating a poor prognosis. His family does not consider allo-SCT, therefore, salvage chemotherapy after relapse of APL is administered, specifically: VEN 100 mg day 4, 200 mg day 5, 400 mg days 6–11; Idarubicin 20 mg days1–2; Cytarabine 100 mg bid days 1–5. The trend of blood cell changes in the patient during treatment is shown in Figure 3.

**Figure 4.**
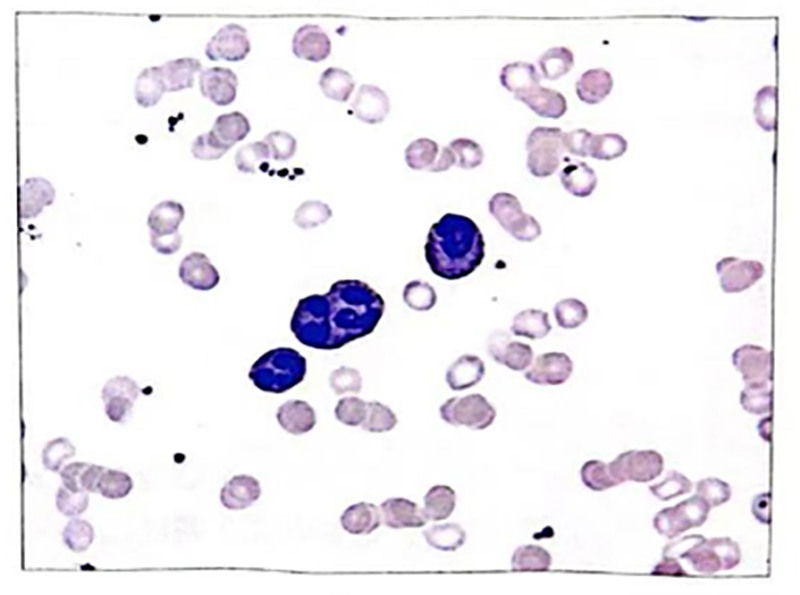
Peripheral blood counts after VEN. VEN = venetoclax.

**Figure 5.**
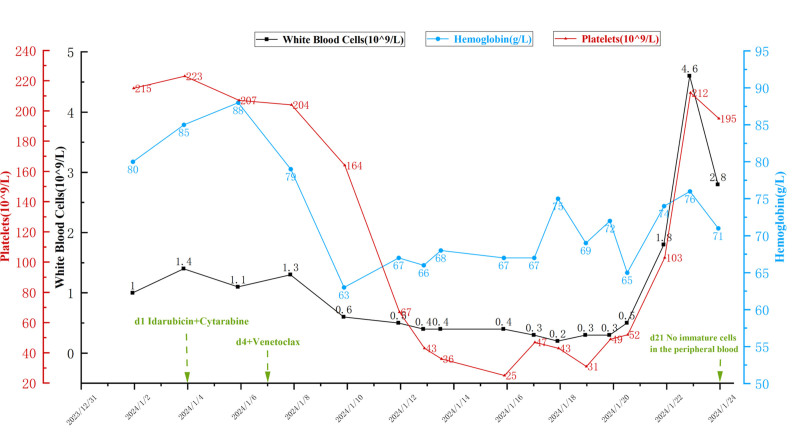
Bone marrow findings after treatment.

**Figure 6.**
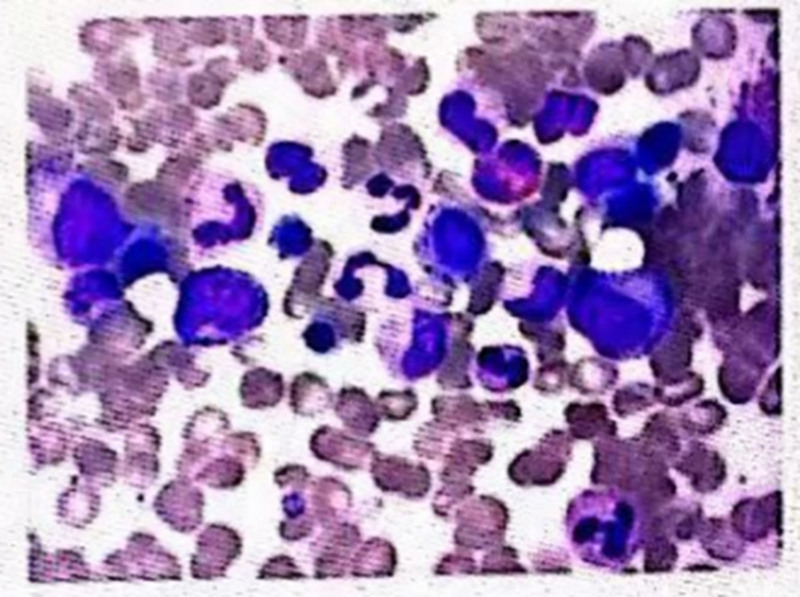
Bone marrow biopsy figure after VEN. VEN = venetoclax.

During chemotherapy, the patient experienced grade 4 myelosuppression and developed an infection. After aggressive treatment with blood product transfusion and targeted anti-infective therapy, the patient’s condition improved. Ten days after the completion of chemotherapy, a peripheral blood smear showed no evidence of immature cells (Fig. 4). Two months post-chemotherapy, a bone marrow biopsy revealed <5% immature cells in the marrow (Figs. 5 and 6). Genetic testing indicated that the PML-RARαL-type (Bcr1 type) was negative. The patient exhibited no symptoms of leukemia and had achieved complete response.

## 3. Discussion

Acute promyelocytic leukemia, as a distinct subtype of AML, accounts for approximately 10% to 15% of confirmed AML cases.^[1]^ The combination of ATRA and ATO, which acts by targeting the PML-RARα fusion protein and thereby inducing promyelocyte differentiation and apoptosis, has become the standard treatment for APL with outstanding therapeutic outcomes. Despite the significant improvement in the cure rate for APL, some patients may still experience relapses, particularly those with atypical APL, or patients with refractory or relapsed APL, where treatment remains a challenge.^[3,5–10]^

For relapsed/refractory APL, the clinical approach often employs ATO combined with intensive chemotherapy as a salvage regimen. ATO combined with idarubicin regimen synergistically enhances antileukemic effects.^[11]^ ATO combined with gemtuzumab ozogamicin (GO) targets CD33 to eradicate minimal residual disease, particularly in individuals with high tumor burden or high CD33 expression.^[12,13]^ For patients harboring FLT3/ITD mutations, the addition of FLT3 inhibitors,such as sorafenib can significantly improve therapeutic outcomes.^[14]^ The patient had experienced multiple relapses and had failed to achieve complete remission after several lines of chemotherapy. Given the reduced functional reserve of vital organs and the results of genetic profiling, VEN-based salvage therapy was selected.

VEN is an oral Bcl-2 inhibitor that targets the Bcl-2 protein to suppress the apoptosis resistance of tumor cells, thereby exerting its anti-tumor effects. Its mechanism of action involves mimicking the function of BH3-only proteins, competitively binding to the Bcl-2 protein, thereby inhibiting Bcl-2’s anti-apoptotic function.^[15,16]^ The Bcl-2 protein is highly expressed in many hematological malignancies, protecting cells from apoptosis by preventing the activation of BAX and BAK proteins. VEN selectively binds to Bcl-2 in the BH3-binding groove, directly and indirectly (by releasing BIM) relieving the inhibition of BAX/BAK, leading to increased mitochondrial permeability, the release of cytochrome C, activation of caspases, and thus inducing apoptosis.^[5]^

Some literature has indicated that when VEN is used in combination with chemotherapeutic drugs such as cytarabine and idarubicin, it may enhance the cytotoxic effects of these drugs.^[17]^ Chemotherapy drugs can damage the DNA and cellular structure of tumor cells, while VEN promotes the apoptotic process in the damaged cells. At the same time, the use of VEN may reduce the resistance of tumor cells to chemotherapeutic drugs, overcoming the defensive mechanisms of tumor cells by promoting the activation of apoptotic pathways.^[5]^

In relapsed/refractory acute promyelocytic leukemia, resistance is frequently attributable to mutations within PML–RARα, most notably those affecting the PML zinc-finger domain that impair ATO binding, or to mutations within the ATRA-binding pocket.^[18]^ Continued use of ATRA/ATO therefore offers limited efficacy in these patients. In many relapsed/refractory APL cases, leukemic blasts evade apoptosis induced by conventional agents through up-regulation of BCL-2. VEN, by selectively inhibiting BCL-2 and functionally mimicking “BH3-only” proteins, directly triggers the mitochondrial apoptotic pathway independent of PML–RARα.^[19]^ This mechanism underlies its capacity to surmount specific resistance pathways.

Although large-scale clinical trials are lacking, an expanding body of case reports and small series has substantiated the potential of VEN in relapsed/refractory APL. A patient with TFG::RARA-variant APL, CD56-positive and of poor prognosis, achieved sustained complete remission after VEN plus ATRA, with an overall survival (OS) > 30 months and progression-free survival of 29 months.^[20]^ Youli Li et al.^[19]^ described a 37-year-old woman who relapsed with an RARA-LBD mutation and reinduction with ATRA and arsenic trioxide combined with anthracycline failed. She attained rapid hematologic remission upon switching to single-agent VEN.

In the treatment of hematologic malignancies, VEN has demonstrated significant efficacy. In AML patients, the combination of the BCL-2 inhibitor VEN with low-intensity therapy has been observed to achieve high response rates and longer duration of response, even in relapsed/refractory settings.^[21–23]^ For patients with relapsed/refractory chronic lymphocytic leukemia, the oral BCL-2 inhibitor VEN is an effective long-term continuous treatment option.^[24,25]^ A case report has stated that a patient with acute undifferentiated leukemia achieved CR after receiving a combination therapy of VEN and azacitidine.^[26]^ These studies suggest that VEN has shown potential therapeutic effects in a variety of hematological malignancies. The combined use of VEN with chemotherapeutic drugs holds promise in the treatment of certain types of hematological malignancies.

VEN is generally considered to have a manageable tolerability profile when treating hematological malignancies, but it may also be associated with some adverse events. The most common adverse events (AEs) include grades 3 to 4 neutropenia, as well as diarrhea, nausea, and Constipation.^[27]^ Additionally, serious adverse events (SAEs) may be observed, such as tumor lysis syndrome, febrile neutropenia, pneumonia, thrombocytopenia, or upper respiratory tract infections.^[27]^ It is important to note that the use of VEN must strictly follow clinical guidance principles, including dose adjustment, efficacy assessment, and management of adverse reactions. The dose of VEN is gradually increased; this dose-escalation plan is designed to reduce the risk of tumor lysis syndrome while allowing the drug to reach a stable concentration in the body to achieve therapeutic effects.^[15]^

This case involves a patient with relapsed/refractory APL who initially experienced multiple relapses after induction therapy with ATO, ATRA, idarubicin, and cytarabine. Subsequent treatment with a combination of ATO, ATRA and anthracycline drugs was unsuccessful. The patient finally achieved both morphological and molecular complete response after salvage chemotherapy with VEN, idarubicin, and cytarabine. During treatment, the patient developed grade 4 myelosuppression, characterized by neutropenia and thrombocytopenia, and concurrent infection with fever. After active transfusion of blood products and symptomatic anti-infective treatment, bone marrow hematopoietic function recovered, and pulmonary infection improved significantly. The patient had a good tolerance to VEN, and the complete hematologic response indicated the significant efficacy of VEN in this case, suggesting that Bcl-2 inhibitors may become an effective salvage therapy for relapsed/refractory APL patients.

## 4. Conclusions

We describe a successful case of a patient with multiply relapsed APL treated with VEN in combination with idarubicin and cytarabine. Although VEN has shown significant efficacy in the treatment of certain hematological malignancies, its application in APL, especially in patients with relapsed/refractory APL, remains relatively rare. Our case indicates that VEN, as a BCL-2 inhibitor, has its unique anti-tumor effects and holds promise to become an effective treatment option for patients with relapsed/refractory APL. Future efforts should identify biomarkers that predict BCL-2 dependency, such as BH3 profiling, to refine the optimal regimen and timing of VEN-based combinations, and to explore its potential to prevent resistance when integrated into earlier lines of therapy.

## Author contributions

**Conceptualization:** Xiaolin Zhang.

**Data curation:** Xiaolin Zhang.

**Funding acquisition:** Chunmei Hu, Rongfeng Qu.

**Investigation:** Xiaolin Zhang.

**Methodology:** Rongfeng Qu.

**Project administration:** Shuhong Hao.

**Resources:** Shuhong Hao, Chunmei Hu, Rongfeng Qu.

**Supervision:** Chunmei Hu, Rongfeng Qu.

**Visualization:** Xiaolin Zhang, Shuhong Hao.

**Writing – original draft:** Xiaolin Zhang.

**Writing – review & editing:** Rongfeng Qu.
